# L-arginine Supplementation Does Not Enhance Anaerobic Performance in Trained Female Handball Players

**DOI:** 10.5114/jhk/197336

**Published:** 2025-07-21

**Authors:** Mozhgan Mardokhi, Mohammad Rahman Rahimi, Saber Saedmocheshi, Manuel Vasquez-Muñoz, David Cristobal Andrade

**Affiliations:** 1 Department of Exercise Physiology, Faculty of Humanities and Social Sciences, University of Kurdistan, Sanandaj, Iran.; 2 Center for Health Data Observation and Analysis (CADS), School of Medicine and Health Sciences, Universidad Mayor, Santiago, Chile.; 3 Exercise Applied Physiology Laboratory, High Altitude Medicine and Physiology Research Center, Biomedical Department, Faculty of Health Science, Antofagasta University, Antofagasta, Chile.

**Keywords:** interval training, anaerobic power, speed, agility, team sports, aerobic capacity

## Abstract

This study aimed to investigate the effect of eight weeks of high-intensity interval training (HIIT) plus L-arginine supplementation on performance of highly trained female handball players. Thirty-two female handball athletes (age: 20.69 ± 0.45 years, body height: 169.38 ± 0.57 cm, body mass: 66.49 ± 1.06 kg) were randomly assigned to a placebo (n = 8), a L-Arg (n = 8), a HIIT+placebo (n = 8) or a HIIT+L-Arg (n = 8) group. HIIT was performed 2 days/week for 8 weeks and consisted of running at 90 to 95% of maximum aerobic speed with 15 s of active recovery, with all training sessions performed on a handball court. The L-arginine supplementation was 0.1 g/kg on training days and 0.05 g/kg on rest days. Performance was assessed using a comprehensive battery of tests, including the 20-m sprint test, the T-agility test, the Cooper test, and the running-based anaerobic sprint test (RAST). Both high-intensity interval training (HIIT) and L-arginine (L-Arg) supplementation led to significant improvements in anaerobic power and 20-m sprint speed (p < 0.05). However, combining HIIT with L-Arg resulted in improvements solely in anaerobic power, without yielding any additional benefits compared to HIIT alone. Notably, all intervention groups (L-Arg, HIIT, and HIIT + L-Arg) experienced significant declines in agility performance (p < 0.05). None of the strategies improved performance during Cooper test. These findings suggest that L-Arg supplementation during HIIT does not confer additional performance benefits and may even exert detrimental effects. Therefore, HIIT alone appears to be sufficient for enhancing anaerobic capacity in highly trained female handball players, and the use of L-Arg supplementation may be unnecessary or counterproductive in this context.

## Introduction

Handball is a collision sport characterized by dynamic movements, including jumping, short accelerations, rapid changes of pace, blocking, pushing, and throwing (Ferrari et al., 2019). As such, handball players require high-intensity training to develop agility, explosive power, speed, aerobic capacity, and coordination (Robilova and Patidinov, 2022). Multiple training protocols have been proposed to optimize performance in handball athletes, such as plyometric training (Ramírez-Campillo et al., 2013; Karpov et al., 2021), neuromuscular interventions (Muñoz et al., 2020), medicine ball exercises (Daneshjoo et al., 2022), handball small-sided games (Bělka et al., 2023), resistance training with elastic bands, and high-intensity interval training (HIIT) (Jurišić et al., 2021).

Among these protocols, HIIT has emerged as one of the most effective methods to enhance performance in handball (Jurišić et al., 2021). HIIT is well-recognized for improving both aerobic and anaerobic capacity, offering tangible benefits to athletes engaged in high-intensity sports (Morais et al., 2025; Seipp et al., 2022). Furthermore, several studies have highlighted its effectiveness in enhancing key performance metrics, including maximal aerobic speed (Agostino, 2019), sprint performance (Germano et al., 2022; Ahmed et al., 2025), the lactate threshold (Esfarjani and Laursen, 2007), and repeated sprint ability (Fernandez-Fernandez et al., 2012)—all critical components for competitive success in handball.

In addition to structured training, diet and supplementation are essential in optimizing athletic performance (Cruzat et al., 2014). Specific supplements aimed at delaying fatigue and improving oxygen delivery to working muscles have been shown to be particularly beneficial (Fielding et al., 2018; Pedlar et al., 2018). Among these, L-arginine (L-Arg), a non-essential amino acid (McConell, 2007; Bailey et al., 2010), has garnered attention for its potential ergogenic effects (Yavuz et al., 2014).

L-Arg supplementation has been explored in several regimens, including long-term (>7 days), short-term (<7 days), and single-dose protocols, with dosages ranging from 0.1 g/kg to 1 g/kg per day, demonstrating positive short-term effects (Hurst et al., 2014; Yavuz et al., 2014). Additionally, long-term supplementation with low (0.03 g/kg) and high doses (12 g/day) over 45 to 56 days has shown improvements in performance among trained adult men (Bailey et al., 2010; Pahlavani et al., 2017). Mechanistically, these enhancements are thought to result from increased secretion of the growth hormone-releasing hormone (GH-RH), suppression of the growth hormone-inhibiting hormone, and elevated levels of the insulin-like growth factor 1 (IGF-1) (Ghigo et al., 1991; Wu and Morris Jr, 1998).

Despite the individual benefits of HIIT and L-Arg supplementation, limited evidence exists regarding their separate and combined effects on anaerobic performance in female handball athletes. Therefore, the present study aimed to investigate the impact of HIIT with and without L-Arg supplementation, on exercise performance of highly trained female handball players.

## Methods

### Participants

Thirty-two young, highly trained female handball players, representing the top team in the first league, voluntarily participated in this study. All athletes belonged to the same club and adhered to similar training regimens before and during the research period. Participants were randomly assigned to one of the four groups: placebo (n = 8; age: 21.15 ± 2.35 years; body mass: 67.30 ± 4.33 kg; body height: 1.70 ± 2.60 m), L-Arg (n = 8; age: 20.88 ± 2.35 years; body mass: 64.30 ± 1.33 kg; body height: 1.67 ± 2.60 m), HIIT+Placebo (n = 8; age: 20.75 ± 1.75 years; body mass: 67.66 ± 8.10 kg; body height: 1.70 ± 4.12 m), and HIIT+L-Arg (n = 8; age: 20.15 ± 1.92 years; body mass: 68.30 ± 5.33 kg; body height: 1.69 ± 2.60 m). All participants had more than five years of handball training experience.

The study employed a single-blind design: while participants were unaware of whether they received L-Arg or a placebo, the researcher responsible for supplementation administration was informed of the container contents. Athletes trained five days per week for approximately 90 min per session, engaging in technical, tactical, strength, and speed exercises identical across all groups. All procedures were conducted during the pre-competitive phase of the annual training cycle.

Participants were screened to ensure they were healthy and free of any supplementation other than the administered L-Arg. Written informed consent was obtained after participants were thoroughly briefed on the study’s purpose, potential risks, and conditions. The research complied with the ethical principles of the Declaration of Helsinki and received approval from the Ethics Committee of the University of Kurdistan (approval number: IR.UOK.REC.1706176; approval date: 19 July 2021). The exercise training protocol is detailed in Table 1.

**Table 1:** Training characteristics.

Rest interval duration between sets	Rest interval duration between repetitions	Intensity of HIIT	Number of repetitions x time	Protocol (HIIT and L-Arg)	Days	Training
3 min 3 min	15 s 15 s	15-s running at 90% of MAS 15-s running at 90% of MAS	2 x 6 min 2 x 6 min	HIIT Handball training HIIT	Saturday Monday Wednesday	week 1
3 min 3 min	15 s 15 s	15-s running at 90% of MAS 15-s running at 90% of MAS	2 x 6 min 30 s 2 x 6min 30 s	HIIT Handball training HIIT	Saturday Monday Wednesday	week 2
3 min 3 min	15 s 15 s	15-s running at 90% of MAS 15 s running at 90% of MAS	2 x 7 min 2 x 7 min	HIIT Handball training HIIT	Saturday Monday Wednesday	week 3
3 min 3 min	15 s 15 s	15-s running at 90% of MAS 15-s running at 90% of MAS	2 x 7 min 30 s 2 x 7 min 30 s	HIIT Handball training HIIT	Saturday Monday Wednesday	week 4
3 min 3 min	15 s 15 s	15-s running at 90% of MAS 15-s running at 90% of MAS	2 x 7 min 30 s 2 x 7 min 30 s	HIIT Handball training HIIT	Saturday Monday Wednesday	week 5
3 min 3 min	15 s 15 s	15-s running at 90% of MAS 15-s running at 90% of MAS	2 x 8 min 15 s 2 x 8 min 15 s	HIIT Handball training HIIT	Saturday Monday Wednesday	week 6
3 min 3 min	15 s 15 s	15-s running at 90% of MAS 15-s running at 90% of MAS	2 x 7 min 30 s 2 x 7 min 30 s	HIIT Handball training HIIT	Saturday Monday Wednesday	week 7
3 min 3 min	15 s 15 s	15-s running at 90% of MAS 15-s running at 90% of MAS	2 x 7 min 15 s 2 x 7 min 15 s	HIIT Handball training HIIT	Saturday Monday Wednesday	week 8

MAS: maximum aerobic speed; HIIT: high intensity interval training

### Measures

The Cooper test was designed to assess the maximum distance covered in 12 min and, in this study, was conducted according to the established procedures (Cooper, 1968). Athletes ran on a 40 × 20 m handball field, as previously described (Krustrup et al., 2003), with validation reported by Penry et al. (2011). Four cones were placed at intervals to demarcate the field dimensions (40 × 20 m). Athletes completed a 5-min warm-up before starting the test. Following a "go" command, participants ran continuously for 12 min within the designated field dimensions.

The running-based anaerobic sprint test (RAST) was used to evaluate anaerobic capacity (Andrade et al., 2015). Two cones were positioned 35 m apart, consistent with the validated methodology (Bongers et al., 2015). After a 5-min warm-up, the time recording began at the examiner’s "go" command. Participants completed six 35-m sprints with 10-s rest intervals in between. Power output for each sprint was calculated using the following formula:


$$Power=\frac{W\cdot D}{t^{3}}$$

where W = body mass, D = distance^2^, and t = time^3^

The 20-m sprint test was conducted indoors on a handball court, adhering to a previously validated protocol (Ramírez-Campillo et al., 2013). Participants initiated the sprint from a crouch start. Infrared beams, positioned 1 m above the floor at a designated distance, were used to measure performance. Athletes completed a warm-up to familiarize themselves with the timing equipment. Each participant performed three trials, and the best result was used for statistical analysis. A 3-min rest interval was allowed between trials. Time was recorded using a photoelectric cell (Globus, Codogne, Italy).

Agility was assessed using the T-agility test, validated by Raya et al. (2013). The test setup involved four cones forming a "T" shape: the first cone (starting point), two side cones marking left and right positions (5 m apart), and a final cone indicating the return point. The test was conducted indoors on a handball court. Participants began in a prone position and started the circuit upon hearing a random auditory signal. Infrared beams, positioned 1 m above the floor at the finish line, measured performance via a photoelectric cell (Globus, Codogne, Italy). Athletes completed two trials, with the best result used for subsequent analysis, and a 3-min rest interval was allowed between attempts.

L-arginine supplementation and the placebo (flour) were administered orally over eight weeks, following a protocol similar to that of Hurst et al. (2014). Participants were blinded to the treatment condition, however, the researcher responsible for supplementation administration was aware of the container contents (single-blind design). On training days, athletes consumed a total of 6 g of supplements: 1 g before breakfast, 2 g 30 min before training, 2 g one hour post-training, and 1 g just before going to sleep (equivalent to 0.1 g/kg). On rest days, participants consumed 3 g of supplements: 2 g before breakfast and 1 g before going to sleep (0.05 g/kg) (Hurst et al., 2014).

### Design and Procedures

Before initiating the intervention, a preparatory meeting was held to familiarize participants with the training program and supplement administration protocols. During this session, participants were introduced to the principles of high-intensity interval training (HIIT) and received detailed instructions on the proper consumption of the placebo and L-arginine (L-Arg) supplements. All assessments were conducted on a handball court under controlled environmental conditions, with temperatures maintained between 19 and 22°C, between 09:00 a.m. and 2:00 p.m. The recorded measures included anthropometric characteristics (body height and mass), results of the running-based anaerobic sprint test (RAST) (expressed in watts), 20-m sprint time (s), the Cooper test results (m), and the T-agility test time (s). These performance tests have been previously validated and widely used in handball athletes (Jurišić et al., 2021).

The intervention spanned eight weeks, with pre- and post-tests conducted following a one-week familiarization period. Participants were instructed to refrain from engaging in strenuous activities for at least two days prior to each testing session.

On handball training days, participants performed a structured 75-min session consisting of 15 min of general exercise (running, jogging, sprinting, jumping, lateral movements, and backward running), followed by 15 min of ball-handling drills, 30 min of sport-specific handball training, and 15 min of static stretching for the cooldown.

Two additional days per week were allocated to the HIIT protocol. These sessions included a 15-min general warm-up, 15 min of ball-based warm-up activities, and approximately 45 min of high-intensity interval training. The HIIT regimen involved running at 95% of maximum aerobic speed with 15-s active recovery intervals, followed by a 15-min cooldown period with static stretching. The training protocol was adapted from the methodology proposed by Krustrup et al. (2003).

### Statistical Analysis

All data are expressed as mean ± standard deviation (SD). The Shapiro-Wilk test was applied to assess the normality of the data distribution, while the Levene’s test was used to verify homoscedasticity of variances. Comparisons between pre- and post-intervention measures for HIIT and L-arginine (L-Arg) administration were conducted using a two-way ANOVA (4 × 2 design) with Holm-Sidak post hoc analysis to identify specific differences between groups. Changes in delta (Δ) effects (post-pre differences) were analyzed separately using a one-way ANOVA, also followed by Holm-Sidak post hoc analysis. Statistical significance was set at an alpha level of *p* < 0.05. All analyses were performed using GraphPad Prism software (version 9.2; GraphPad Software, La Jolla, CA, USA).

## Results

**Figure 1:**
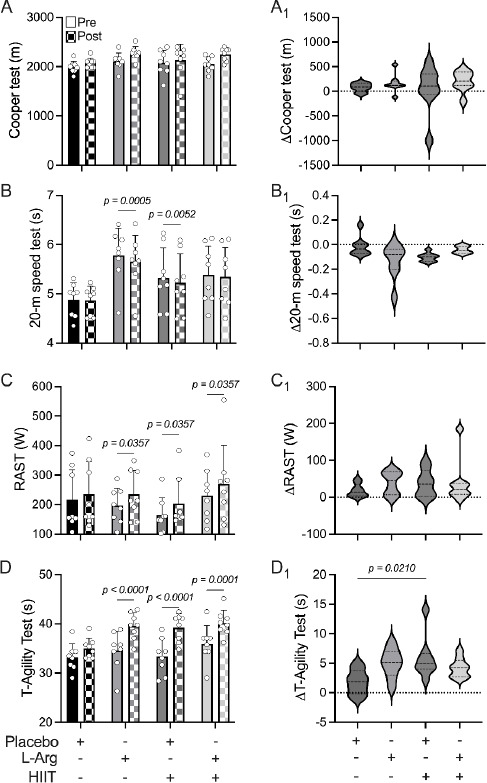
Effects of 8 weeks of high-intensity interval training (HIIT) and exogenous L-Arg administration on exercise performance. A) Effect of L-Arg, HIIT, and their combination on Cooper test performance. A1) The pre-post effect was not different between groups. B) In a single-blind design, eight weeks of HIIT and L-Arg improved maximum speed during a 20-m speed test, which was blunted when combined. B1) The pre-post effect was not different between groups. C) HIIT, L-Arg, and HIIT-L-Arg improved anaerobic power which was determined through the running-based anaerobic sprint test (RAST). C1) The pre-post effect was not different among groups. D) Agility deteriorated with all protocols (HIIT, L-Arg, and HIIT+L-Arg). D1) Agility performance declined following the HIIT protocol compared to the placebo condition. Values are mean ± SD, n = 8 per group.

Our results indicated that neither L-arginine (L-Arg) supplementation nor high-intensity interval training (HIIT), alone or combined, significantly affected maximal aerobic performance in female handball players (Figure 1A). The change in delta (Δ) effect (post-pre) was not significantly different between groups (Figure 1A1).

Notably, eight weeks of L-Arg supplementation and HIIT demonstrated a significant improvement in maximal speed (Figure 1B). Specifically, L-Arg (5.64 ± 0.53 vs. 5.78 ± 0.55 s, Post vs. Pre, respectively, Figure 1B) and HIIT (5.22 ± 0.59 vs. 5.33 ± 0.61 s, Post vs. Pre, respectively, Figure 1B) significantly reduced execution time during the 20-m speed test. However, L-Arg administration appeared to promote an interference effect with adaptations induced by HIIT training (5.34 ± 0.60 vs. 5.38 ± 0.58 s, Post vs. Pre, respectively, *p* > 0.05, Figure 1B). Furthermore, the Δ effect (post-pre) was not significantly different between groups (Figure 1B1).

In addition to the 20-m speed test, our data revealed that L-Arg (234.64 ± 81.36 vs. 195.86 ± 60.03 W, Post vs. Pre, respectively, Figure 1C), HIIT (203.32 ± 85.38 vs. 164.66 ± 58.96 W, Post vs. Pre, respectively, Figure 1C), and the combined HIIT regimen with L-Arg supplementation (269.77 ± 131.12 vs. 230.55 ± 86.16 W, Post vs. Pre, respectively, Figure 1C) significantly increased power output in highly trained handball players. Nevertheless, the Δ effect (post-pre) remained consistent across groups (Figure 1C1).

Although our data demonstrated that L-Arg, HIIT, and the combined HIIT+L-Arg interventions yielded significant improvements in anaerobic performance, agility was not similarly affected. The results indicated a decline in agility across all groups, potentially reflecting neuromuscular fatigue or limitations in recovery protocols (Figure 1D, all *p* < 0.001). Additionally, the Δ effect (post-pre) was significantly different between the Placebo and HIIT+Placebo groups (Figure 1D1).

## Discussion

The main aim of the present study was to investigate the effect of HIIT with and without L-Arg supplementation on exercise performance of highly trained female handball athletes using a single-blind study design. Our findings revealed that: i) eight weeks of either a HIIT regimen or L-Arginine supplementation significantly improved maximum speed, as demonstrated by the 20-m speed test results, and anaerobic power, assessed through the running-based anaerobic sprint test (RAST); ii) HIIT combined with L-Arginine supplementation resulted in significant improvement in anaerobic power only; iii) L-Arg, HIIT, and HIIT+L-Arg all led to a decline in agility performance; and iv) neither HIIT, L-Arg nor their combination affected performance during the Cooper test. These results suggest that HIIT and L-Arg enhance anaerobic capacity independently, but no additional benefits were observed when combined. This finding highlights a potential interference effect between HIIT and L-Arg administration in female handball players.

The combined effect of HIIT and L-Arg supplementation on running speed is likely due to increased glycolytic enzyme activity and nitric oxide pathways. However, interference effects between the two may hinder expected gains in some performance metrics (Bailey et al., 2010; Fernandez-Fernandez et al., 2012; Jurišić et al., 2021). This improvement might be associated with increased glycolytic enzyme activity promoted by the HIIT regimen (Pahlavani et al., 2017). Naimo et al. (2014) demonstrated that HIIT positively influenced power output, improving anaerobic performance in hockey players. Similarly, Fajrin et al. (2018) reported that HIIT enhanced explosive strength and 20-m sprint test performance in basketball players, attributed to improved biomechanical properties. Explosive movements, such as those performed during HIIT, may facilitate elastic energy accumulation during the eccentric contraction phase, which is subsequently utilized during the concentric phase. Thus, HIIT likely increases elastic energy storage and efficiency during the 20-m sprint test, partially explaining our results.

Our data also revealed that L-Arg administration could enhance anaerobic performance. Previous studies have explored the impact of L-Arg supplementation on athletic performance, reporting improvements in sprint performance (Buford and Koch, 2004), time to exhaustion during high-intensity cycling (Bailey et al., 2010), and muscle strength (Campbell et al., 2004). Campbell et al. (2006) further demonstrated the positive effects of L-Arg supplementation on anaerobic performance, including maximum weight repetition and Wingate test outcomes. However, Birol et al. (2019) observed that L-Arg supplementation did not improve speed or muscle endurance (Greer and Jones, 2011). Although this study utilized 6 g/day (0.1 g/kg body weight), evidence suggests that the optimal dosage and scheduling have not been yet universally defined (Abel et al., 2005; Birol et al., 2019). Future research should evaluate the effects of multiple L-Arg dosing protocols on exercise performance.

In contrast to anaerobic performance, our findings indicate that aerobic performance did not improve and even deteriorated after eight weeks of L-Arg supplementation, contradicting existing evidence. L-Arg supplementation has been associated with enhanced aerobic performance, attributed to nitric oxide-related blood flow, increased oxygen uptake, and reduced lactate accumulation (Campbell et al., 2004; Jones et al., 2016; Tang et al., 2011; Zajac et al., 2010). However, the use of 6 g/day in this study might have been insufficient, as doses exceeding 10 g/day are recommended for greater effectiveness. Additionally, timing of consumption may play a critical role. Evidence suggests that consuming L-Arg three hours before exercise optimizes nitric oxide-related blood flow (Bonilla Ocampo et al., 2018; Bode-Böger et al., 1998). Thus, although L-Arg is expected to improve aerobic performance via nitric oxide pathways, our results suggest that dose and timing limitations may have contributed to the observed deterioration in aerobic capacity. Further research is required to explore these mechanisms in highly trained handball players.

This study, like others in the field, has some limitations. First, the single-blind design may have introduced potential bias, as a double-blind design would provide stronger reliability. Second, the exclusive inclusion of female participants without a male comparison group limits generalizability and could influence conclusions. Third, the 6 g/day dose used in this study was lower than the recommended 10 g/day for significant effects. Furthermore, this study relied on indirect performance assessments, which, while commonly used by scientists and coaches, may lack precision. Biochemical variables, such as nitric oxide plasma levels, were also not measured, leaving physiological mechanisms unexplored. Lastly, a wider range of L-Arg doses and interval training protocols should be investigated in future studies.

## Conclusions

Our results strongly suggest that L-Arg supplementation during a HIIT regimen does not provide additional benefits compared to the separately observed effects. It may even promote disadvantageous effects, suggesting that HIIT itself could improve anaerobic performance in female professional handball athletes without needing L-Arg administration during the training regimen.
